# Comment regarding: ‘On the solubility of azodicarbonamide in water/DMSO mixtures: an experimental and computational study’ (2024), by Macetti *et al.*

**DOI:** 10.1098/rsos.241011

**Published:** 2024-09-11

**Authors:** Josje Arts, Ian Kimber

**Affiliations:** ^1^ Nouryon, Zutphenseweg 10, 7418 AJ Deventer, The Netherlands; ^2^ Faculty of Biology, Medicine and Health, University of Manchester, Manchester M13 9PL, UK

**Keywords:** respiratory allergy, respiratory sensitization, ADCA

According to Macetti *et al*. [1], in the Introduction section of their recent paper, reference is made to two published papers as follows: *Even though ADCA is still employed in the production of foamed plastics* [2]*, it was classified as a respiratory sensitizing agent* (*H334: May cause allergy or asthma symptoms or breathing difficulties if inhaled*) *as for CLP* (*Classification, Labelling and Packaging*) *Regulation* (*EC*) *No. 1272/2008, generating concerns especially at chemical industries where it is manufactured and used* [3].

In our paper [2], we stated that although azodicarbonamide (ADCA, CAS no. 123-77-3) had been officially classified as a respiratory sensitizer, the purpose of our article was to consider whether this classification is appropriate based on clinical experience and relevant experimental data. We concluded that although there have been reports of an association between workplace exposure to ADCA and respiratory symptoms and occupational asthma, the evidence is considerably less than persuasive, with in many instances there being a lack of properly controlled and executed diagnostic procedures. In addition, ADCA fails to elicit positive responses in mouse and guinea pig predictive tests for skin sensitization—a lack of activity that is regarded as being inconsistent with respect to respiratory sensitizing potential. In addition, the German MAK Commission [4] indicated with respect to ADCA that the decisive factor for the assessment of a possible sensitizing potential is that none of the studies reported the origin, identity and purity of the substance that was causative at the workplace or used for diagnostics and that, in view of the numerous potential additives present during the production or use of ADCA, the substance is not clearly identifiable as the cause of the effects. Moreover, available results on metabolism and considerations on the reactivity and mode of action of the compound do not clearly point to protein binding *in vivo,* which would be a prerequisite for a sensitizing potential on the skin or airways, and thus the available data are not sufficient to demonstrate sensitizing effects for ADCA.

Collectively, the data reviewed in the paper by Arts & Kimber [2] and cited as reference number 4 by Macetti *et al*. in their recent paper [1] do not provide a sound basis for the classification of ADCA as a respiratory allergen.

Indeed, ADCA is widely used by industry in the manufacture of a variety of products, and it was noted [2] that, with only a single exception, associations between workplace exposure to ADCA and symptoms of respiratory allergy were reported before 2000, and in only three cases [5,6] were the symptoms and clinical investigations well documented. In addition, according to the MAK Commission [4], although ADCA has also been used as a blowing agent in Germany for a long time, no cases of sensitization have been observed.

It is, therefore, highly remarkable that Macetti *et al*. [1] are drawing inappropriate conclusions based upon a paper [3] published a quarter of a century ago.

## Data Availability

This article has no additional data.
